# Epidemiological Aspects of Exotic Malaria and Dengue Fever in Travelers in Korea

**DOI:** 10.4021/jocmr571e

**Published:** 2011-05-19

**Authors:** Sang-Hee Park, Myeong-Jin Lee, Jun-Hee Baek, Won-Chang Lee

**Affiliations:** a Division of Zoonoses, Center of Immunology and Pathology, Korea Center of Disease Control and Prevention, Korea; b Public Health in Department of Nutritional Sciences, Otemae College of Nutrition, Osaka, Japan; c University of Maryland, College of Park, Biology Major, USA; d College of Veterinary Medicine, Konkuk University, Seoul (143-701), Korea

## Abstract

In order to compare the epidemiological aspects of exotic malaria (EM) and dengue fever (DF) imported by travelers in Korea, we have analyzed the current state both of the disease incidence and related risk factors. A total of 345 cases of EM occurred between 2001 and 2008 in Korea, and the average incidence rate per 100,000 population was 0.091. A total of 252 of DF cases occurred during the same period, and its rate was 0.063. While most of the EM and DF prevalence occurred in summer, prevalence in spring and winter was more prominent for EM (P < 0.05 ~ P < 0.01), while outbreaks in summer were more frequent for DF (P < 0.01). In Korea, more males were infected with EM and DF than females (P < 0.01). The remarkable difference between gender distributions in Korea is believed to reflect cultural differences in terms of work and travel. In both diseases, the manhood age bracket (20 - 39 years old) is possible due to increased oversea activities and travel. Moreover, reported EM cases in several prefectures in the regions of Asia and Africa were widely spread by the appropriate vector of mosquitoes, while the vectors of DF in the region of Asia are limited.

**Keywords:**

Epidemiology; Exotic malaria; Dengue fever; Traveler; Korea

## Introduction

Numerous diseases are transmitted by insect vectors. Most are restricted to the tropic and only seen in more temperate regions as imported diseases, because of the requirement of certain arthropod vectors like the Anopheles or Aedes mosquitoes [1]. Several vector-borne tropical diseases are protozoa infections, caused by protozoan parasites which replicate and multiply rapidly inside the host. By far the most important vector-borne protozoa infection of malaria is the most lethal protozoan disease and a dominant public health concern in more than 100 nations, and is caused by infection with one or more of four species of *Plasmodium* (i.e., *P. vivax, P. falciparum, P. ovale, and P. malariae*) that can infect humans, and other *Plasmodium* species infect animals. The infection is transmitted by the bite of an infected female *Anopheles* mosquito [2-6]. On the other hand, a great many viruses also are transmitted by arthropod bites, mostly those by mosquitoes or ticks, and for that reason fall under the generic name of “arboviruses”. One of these viruses belongs to the Flaviviridae family, which includes the four dengue fever viruses that are transmitted by mosquitoes. Not surprisingly, arboviruses can cause striking epidemic numbers of cases of illness or deaths. Dengue fever (DF), the most prevalent mosquito-borne viral disease, is estimated to cause 100 million infections each year, 250,000 - 500,000 of which are the causes of severe illnesses [1, 7].

In Korea, no domestic case of malaria has been reported from 1980 to 1992. However, after 1993, with the infection of military personnel in demilitarization (DMZ) area, a number of malaria cases have begun to emerge, and *P. vivax* is the most common type of malaria, prevalent in 97.5% of domestic cases [4]. Moreover, a new exotic malaria (EM) imported by travelers has been increasingly seen in Korea since 1980s. Recently, imported EM and DF have been an increasing problem in Korea in the last two decades, representing the main risk for travelers visiting tropical and sub-tropical countries where malaria and dengue are endemic [5, 8, 9].

In the present descriptive study, we investigated the current epidemiological aspects of EM and DF imported by travelers in Korea, and compared the risk factors between the two diseases.

## Methods

The data on confirmed EM and DF cases in Korea were obtained from the Annual Report of Notifiable Infectious Diseases and Communicable Disease (2001 - 2008) [8], and Public Health Weekly Report (2008) [9], Korea Center for Disease Control and Prevention (KCDCP), Republic of Korea. To better quantify the impact of EM and DF on health in Korea, we compiled and analyzed information including incidence rate and related risk factors including cases of seasonal outbreaks and gender, age distribution and suspected area by travelers. The statistical methods of data analysis included incidence rate (IR) of EM and DF per 100,000 populations and the Chi-square test, which was used to compare the frequency distribution by seasonal, gender, age and cases of suspected areas, and estimating 95% confidence intervals (95% CI). Results were considered to be statistically significant at P < 0.05 and P < 0.01.

## Results and Discussion

Table 1 shows the incidence of EM and DF in Korea from 2001 to 2008. For EM, the average incidence rate between 2001 and 2008 was 0.091 per 100,000 population with a variance from 0.06 to 0.144. During the same period, the incidence rate of DF showed an average of 0.067 with a variation of 0.013 to 0.101. The incidence of EM decreased in this period, while that of DF increased in Korea. The decrease in EM malaria might be attributable to more widespread prevention measures using several methods, such as chemoprophylaxis [10-12], while the rise in DF might be associated with increased opportunities for infection due to the spread of endemic areas worldwide and with the rising coverage of diagnosis due to the enhanced awareness of this disease by physicians [12, 13].

**Table 1:** Comparative Observation of Epidemiological Aspects of Exotic Malaria and Dengue Fever Imported by Travelers in Korea (2001 - 2008)

	Exotic Malaria		Dengue Fever	
	Cases (Rate)	95% CI	Cases (Rate)	95% CI
Total (I.R.)	345 (0.091)	0.081 - 0.101	262 (0.063)	0.055 - 0.071
Season				
Spring	73 (24.3)*	17.5 - 26.3	36 (14.2)	9.9 - 18.5
Summer	118 (35.3)	30.2 - 40.4	125 (49.2)**	43.1 - 55.4
Autumn	62 (18.6)	14.4 - 22.7	55 (21.6)	16.5 - 26.7
Winter	81 (24.3)**	19.7 - 28.9	38 (15.0)	10.6 - 19.4
Total	334 (100%)		254 (100%)	
Gender				
Male	259 (76.2)**	71.7 - 80.7	171 (65.3)**	59.5 - 71.1
Female	81 (23.8)	19.3 - 28.3	91 (34.7)	28.9 - 40.5
Total	340 (100%)		262 (100%)	
Age				
< 19	17 (5.1)	2.8 - 7.4	20 (8.2)	4.7 - 11.7
20 - 29	115 (34.5)	29.4 - 39.6	81 (33.3)	27.4 - 39.2
30 - 39	82 (24.6)	20.0 - 29.2	70 (28.8)	23.1 - 34.5
40 - 49	65 (19.5)	15.2 - 23.8	46 (18.9)	14.0 - 23.8
50 - 59	38 (11.4)	8.0 - 14.8	17 (7.0)	3.8 - 10.2
> 60	16 (4.8)	2.5 - 7.1	9 (3.7)	1.3 - 6.1
Total	333 (100%)		243 (100%)	

IR: incidence rate per 100,000 population (2001 - 2008); 95% CI: confidence intervals of 95% of rate. Chi-square analysis indicated a significant difference from the total value. *P < 0.05, **P < 0.01.

Both EM and DF cases occurred most frequently in summer (35.3% and 49.2% of each total cases); however, the rate of EM outbreaks was relatively higher in spring (24.3%) (P < 0.05) and winter (24.3%) (P < 0.01), while outbreaks in DF were relatively higher in summer (49.2%) (P < 0.01). It is well known that EM and DF infections are affected by seasonal or climatic conditions [1, 9].

As shown in Table 1, significantly more males (76.2% of total cases) were infected by EM than females (23.8%) (P < 0.01), while the number of males infected by DF (65.3% of total cases) was higher than the respective number of females (34.7%) (P < 0.01). These remarkable differences in gender distribution are believed to reflect travel differences between males and females in terms of visiting areas due to work, business, and other activities [9].

The percentage distribution of EM cases by age group was as following: for the age groups of under 19, 20 - 29, 30 - 39, 40 - 49, 50 - 59 and over 60 years, the percentages were 5.1, 34.5, 24.6, 19.5, 11.4, and 4.8%, respectively. The distributions by age group were similar in EM and DF, and over 61.2% of the cases involving DF were in the 20 - 39 years old age bracket, clearly showing a higher incidence rate in men. The constantly mounting movement of travelers from developed countries to the tropics or subtropics and the affluent immigration to the industrialized world from countries where EM and DF have remained are responsible for the emergence of imported EM and DF. Recently, imported EM and DF have been an increasing problem in Korea in the last two decades, representing the main risk for travelers visiting tropical and subtropical countries where EM and DF are endemic [1, 7].

**Table 2:** Comparative Observation on the Reported Cases of Exotic Malaria and Dengue Fever in Travelers in Korea to the Suspected Areas (2001 - 2008)

Region	Exotic Malaria		Dengue Fever	
	Cases	%	Cases	%
ASIA	163	47.9	247	98.0**
China	14	4.1	5	2.0
Thailand	6	1.8	42	16.7**
Philippines	15	4.4	82	32.5**
Cambodia	5	1.5	26	10.3**
Vietnam	2	0.6	16	6.4**
Indonesia	31	9.1	26	10.3
India	36	10.6	20	7.9
Others	54	15.6	30	11.9
AFRICA	154	45.3**	3	1.2
OCEANIA	15	4.4	0	0
Unknown	8	2.4	2	0.8
Total	340	100%	252	100%

Chi-square analysis indicated a significant difference from the total value. *P < 0.05, **P < 0.01.

Table 2 shows comparative observations of the reported cases of EM and DF by travelers in the suspected areas in Korea during the period of 2001 to 2008. In the case of EM, a 47.5% of all EM (340 cases) in Asia region had relations to China (4.1%), Thailand (1.8%), Philippines (4.4%), Cambodia (1.5%), Vietnam (0.6%), Indonesia (9.1%), India (10.6%) and others (15.5%). And 45.3% of all EM cases were related to Africa region, 4.4% to Oceania region, and 2.4% to unknown cases. During the same period, 98.0% of all DF (252 cases) in Asia region had relations to China (2.0%), Thailand (16.7%), Philippines (32.5%), Cambodia (10.3%), Vietnam (6.4%), Indonesia (1.3%), India (7.9%) and others (11.9%). And 1.2% of all DF cases were related to Africa region, and 0.8% to unknown cases. Reported EM cases in several prefectures in the regions of Asia and Africa were widely spread by the appropriate vector of mosquitoes, while those affected by DF in the region of Asia were limited.

Moreover, according to the reported cases of imported malaria by KCDCP, 40.9% of total 303 cases were infected with *plasmodium falciparum*, while 36.0% were diagnosed with *P. vivax* infection, 3.3% with *P. falciparum* and *P. vivax* mixed, 2.3% with *P.malariae*, 1.3% with *P. ovale* and 16.2% with unknowns [8].

Mosquito-borne diseases, such as EM and DF, have a major impact on public health all over the world (WHO, 2011). There are disease-specific characteristics in geographic distribution, temporal trends and seasonality of such cases because their transmission is dependent on the spread and density of the appropriate vector of mosquitoes [1, 13].

The majority of Koreans travel to developing worlds to visit, sightsee, or conduct business in other areas of Asian countries for a short term; EM and DF risks are limited in these cases. Nevertheless, the number of those visiting highly malaria-endemic areas like sub-Saharan Africa or Oceania is steadily increasing. Consequently, countermeasures against a possible increase in the number of EM and DF cases, especially against DF cases in the future, should become a priority [10, 13, 14].

In conclusion, many Korean travelers are unaware of diseases in the countries they visit, and do not adequately protect themselves. Applying carefulness to avoid contact with mosquitoes is very important to avoid EM and DF infection. Nevertheless, this is difficult to do, and practical method of immunization is available to date. Various types of chemoprophylaxis agents for malaria have been used for a long time but not for DF yet. To implement effective education measures against imported EM and DF, an evaluation of infection risks based on the incidence rates of EM and DF per given number of travelers to each area/country is necessary.
